# The Association of Complements, TGF-*β*, and IL-6 with Disease Activity, Renal Damage, and Hematological Activity in Patients with Naïve SLE

**DOI:** 10.1155/2022/7168935

**Published:** 2022-11-08

**Authors:** Yuliasih Yuliasih, Lita Diah Rahmawati, Nabilatun Nisa', Cahaya Prastayudha

**Affiliations:** ^1^Rheumatology Division of Internal Medicine Department, Faculty of Medicine, Airlangga University, Surabaya 60132, Indonesia; ^2^Department of Biology, Faculty of Science and Technology, Airlangga University, Surabaya 60115, Indonesia; ^3^Faculty of Medicine, Airlangga University, Surabaya 60132, Indonesia

## Abstract

Several key player factors, such as cytokine and complement, play an important role in the pathogenesis of systemic lupus erythematosus (SLE). The purpose of this study was to reveal the association between complement 3 (C3), complement 4 (C4), interleukin-6 (IL-6), and transforming growth factor-*β* (TGF-*β*) with SLE disease activity, renal damage, and hematological activity in patients with naïve SLE. The Laboratory of Clinical Pathology Dr. Soetomo General Hospital in Surabaya performed all laboratory examinations on thirty women with naïve SLE. The SLE diagnosis is based on ACR criteria (1998 revised criteria) from Dr. Soetomo General Hospital Surabaya, Indonesia, and the systemic lupus activity measurement (SLAM) score is used to assess the disease activity. The correlation was statistically tested using the Spearman and Pearson tests. The differences in cytokine and complement levels are between SLE severity groups using the two-way Anova and Kruskal–Wallis. The unpaired *T*-test and Mann–Whitney test were used to determine the differences between the relatively normal and the more severe groups of organ damage and hematological activity. All tests were two-tailed, analyzed with GraphPad Prism 9 for windows, and a *p* value of less than 0.05 was considered statistically significant. This study found a significant decrease in C3 (20.2, 16.4–24.2 mg/dL) and C4 (7, 6–14.3 mg/dL) and an increase in IL-6 (35.60 ± 7.43 mg/dL) and TGF-*β* (311.1 ± 290.8 mg/dL) in the group of severe patients with SLAM scores >30. Although there is no significant relationship between SLAM and renal impairment or hematologic activity, patients with higher SLAM had a significant decrease in complement; this complement decrease was also significant in patients with higher leukocyte counts. An insignificant increase in cytokines was also observed in patients with higher SLAM. Patients with high serum creatinine levels had a significant increase in TGF-*β*, whereas those with a faster ESR had a significant increase in IL-6. In conjunction with complements evaluation, assessment of the cytokine profile may become a promising marker for reliable diagnosis and treatment of SLE in the future.

## 1. Introduction

Systemic lupus erythematosus (SLE) is a chronic autoimmune disease characterized by a series of immunological abnormalities and the production of autoantibodies, causing widespread inflammation and leading to tissue and organ damage [1, 2]. Around 40–50 percent of all patients with SLE have significant morbidity and a poor prognosis, with a higher incidence rate and severity of clinical manifestations in Asians and people of African descent but less in Caucasians [3]. Measuring organ damage in patients with SLE is essential because it allows for the development of new treatments that improve disease control while minimizing the development of irreversible damage [4]. The kidney appeared to be the most commonly involved organ, with 60 percent of patients with lupus developing clinically relevant nephritis at some point during the disease [5]. Hematologic abnormalities are also common in patients with SLE and frequently become the diseases' presenting manifestation [6]. Early detection and treatment of renal and hematological disorders are essential because an early response to treatment correlates with better outcomes, emphasizing the importance of regular screening for early and appropriate management.

Several key player factors, such as cytokine overproduction and decreased complement, play an important role in the pathogenesis of SLE. The circulatory system and the endogenous immune complex influence complement, causing infiltration of inflammatory cells, activation of coagulation factors, and release of inflammatory mediators, eventually leading to organ damage, including renal [7]. Patients who lack the early components of the classical complement pathway, including complement 3 (C3) and complement 4 (C4), often exhibit clinical renal SLE symptoms in addition to susceptibility to infection, indicating the development of immunosuppressive effects of SLE [8, 9].

Transforming growth factor (TGF)-*β* and interleukin-6 (IL-6) cytokines can interact to form complex networks that regulate CD4+ T cell responses. The TGF-*β* and IL-6 induce differentiation of pathogenic IL-17, producing T-helper 17 (Th17) subsets that may promote inflammation and enhance autoimmune disease. On the other hand, the absence of IL-6 made TGF-*β* convert naïve Th17 cells to Foxp3-positive regulatory T cells (Tregs) that are essential in suppressing the immune response [10–14]. In addition, higher production of TGF-*β* in patients with SLE may be associated with TGF-*β* polymorphisms that affect its transcription and can cause interindividual variability in cytokine production [15].

Despite numerous reported associations between complement and cytokine in patients with SLE, few studies link C3 and C4 complement, as well as TGF and Il-6 cytokines, to disease activity, renal damage, and hematological activity simultaneously. High racial linkage of complements and cytokines allows disease manifestation and progression differences. In contrast, no studies have been conducted on patients with naïve SLE in Indonesia, particularly at Dr. Soetomo Hospital. The purpose of this study was to reveal the association between C3 and C4 complements, IL-6, and TGF-*β* cytokines with SLE disease activity, renal damage, and hematological activity in patients with SLE. A better understanding by comparing cytokines and complements with SLE activity, renal impairment, and blood activity aims to improve SLE diagnosis and monitoring of the disease and prognosis.

## 2. Materials and Methods

### 2.1. Patients and Study Design

This cross-sectional study involved thirty female patients aged between sixteen to sixty years old with naïve SLE. The diagnosis of SLE is based on ACR criteria (1998 revised criteria) from Dr. Soetomo General Hospital Surabaya, Indonesia. The study was approved by Dr. Soetomo hospital's Research Ethics Committee (Number: 155/Panke.KKE/III/2017), and informed consent was obtained from all patients. The Scientific Advisory Patients of the Department of Internal Medicine underwent a full clinical assessment, including measurement of disease activity using the systemic lupus activity measurement (SLAM) score, the clinical evaluation, and autoantibody profiles. No flare was indicated by a SLAM score <3; 3–12 was moderate activity; and >12 was severe.

### 2.2. Laboratory Assessments

The Laboratory of Clinical Pathology at Dr. Soetomo General Hospital in Surabaya performed laboratory examinations on all subjects. Fresh blood from the antecubital vein of each subject was collected in a serum separator tube and centrifuged for 15 minutes at 1000*g* at 2–8°C. The supernatant was collected into new tubes and stored at −70°C. The IL-6 was duplicated for each subject using a human enzyme-linked immunosorbent assay (ELISA) kit as the manufacturer's protocol (Elabscience, catalog. E-EL-H0102). The hospital laboratory measured serum blood urea nitrogen (BUN) levels, creatinine levels, and proteinuria concentrations as markers of renal impairment. According to the National Kidney Foundation's clinical classifications [16], urine protein concentrations of 30–99 mg/dL are classified as “1+,” 100–299 mg/dL as “2+,” 300–999 mg/dL as “3+,” and 1000 mg/dL as “4+.” The evaluation of complete blood cell count, sedimentation rate, and CRP was also conducted at the hospital laboratory for hematological activity parameters.

### 2.3. Statistical Analysis

The analysis aimed to determine the association of complement and cytokines with renal damage and hematological activity in patients with SLE. The association of the SLAM score with renal impairment and hematological activity was analyzed using Pearson's correlation test (for normal-distributed data) and Spearman's correlation test (for nonformal distributed data). To compare the complements and cytokines in patients with SLE, the SLAM score was categorized into three groups with a range of 20–25, 26–30, and >30 for segregating patients by severity of SLE activity. The comparison of complements level and cytokine levels with the SLAM score were analyzed with a two-way ANOVA for normally distributed data or Kruskal–Wallis for nonformal distributed data. The patients were divided into two groups based on the severity grade of each parameter (BUN, creatinine, leukocyte count, platelet count, hematocrit, and ESR) for comparisons of complements and cytokines levels with renal damage and hematological activity. A statistical analysis was conducted with the unpaired *T*-test (normally distributed data) or Mann–Whitney test (nonformal distributed data). All tests were two-tailed, analyzed with GraphPad Prism 9 for Windows, and a *p* value of less than 0.05 was considered statistically significant.

## 3. Results

### 3.1. Patient's Characteristics

There were 30 women patients at the Dr. Soetomo General Hospital in Surabaya with naïve SLE who were the subject of this study.  Table 1 shows the characteristics and general information of clinical and laboratory results.

### 3.2. Association of Disease Activity with Renal Damage and Hematological Activity

As shown in Table 2, the association between SLE activity, as indicated by the SLAM score, with renal damage and hematological activity was weak. The low value and narrow to 0 of *r* show a weak correlation. In addition, all *p* values were above 0.05, which exhibited no significant relationship. A positive *r*-value represents a positive correlation. On the other hand, a negative value indicates a negative correlation. Therefore, when the SLAM score increased, the BUN, creatinine, leukocyte, platelet, and hematocrit levels also increased, while the urinary protein and ESR decreased.

### 3.3. Comparison of Disease Activity with Complement and Cytokine Levels

**Figure 1:**
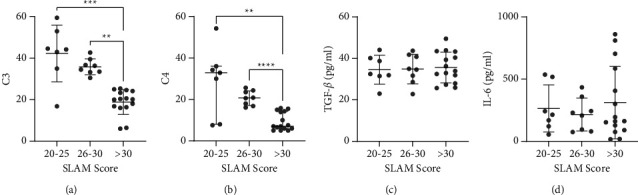
Comparison of complements and cytokines levels among groups of patients with SLE based on their SLAM scores. The *n* of each group was: 20–25 = 7, 26–30 = 8, and >30 = 15. SLAM: systemic lupus activity measurement, C3: complement 3, C4: complement 4, TGF-*β*: transforming growth factor-beta, and IL: interleukin.

There were three groups of SLAM scores, with the larger value indicating the severity of SLE in patients. Significant decreases in the complement level of patients, as shown in Figures 1(a) and 1(b), and are in line with the SLE severity. The C3 levels in the SLAM score of 20–25; 26–30; and >30 were 42.3 ± 13.68 mg/dL, 35.89 ± 3.849 mg/dL, and 20.2 (16.4–24.2) mg/dL, respectively. The mean C4 levels for the group with a SLAM score of 20–25 were 29.00 ± 16.49 mg/dL, for the 26–30 group were 20.74 ± 3.570 mg/dL, and the median of SLAM scores of >30 group was 7 (6–14.3) mg/dL.

There was no significant difference in TGF-*β* and IL-6 levels among all SLAM groups. However, there was an increasing pattern of TGF-*β* following the SLAM scores with mean at 20–25; 26–30; and >30 groups were 34.53 ± 7 mg/dL, 34.85 ± 7.14 mg/dL, and 35.60 ± 7.43 mg/dL, respectively. For IL-6 levels, a decrease occurred in the SLAM group of 20–25 to 25–30 with a mean of 264.8 ± 189.3 mg/dL and 214.8 ± 132.1 mg/dL, respectively. Then, IL-6 for the group with SLAM scores >30 decreased with an average of 311.1 ± 290.8 mg/dL.

### 3.4. Association of Complement and Cytokine Levels with Renal Damage and Hematological Activity

A significant decrease in complements and elevated cytokines was found in the more severe patients. Patients with leukocytosis significantly decreased complement levels, while the TGF-*β* and IL-6 significantly increased in patients with high creatinine levels and higher ESR (Table 3). Despite being statistically insignificant in other parameters, increased cytokine levels occur in patients with more severe renal damage and hematological activity. In contrast, the complements tend to experience an insignificant increase in patients with higher BUN and creatinine levels but a decrease in patients with higher hematological activity.

## 4. Discussion

The SLE disease primarily affects women, as evidenced by our study, which found that all patients with SLE at Dr. Soetomo General Hospital were female. Concordant with Megan et al.'s [17] review, which summarizes that the female lupus population in Asia, particularly Taiwan, Korea, China, and the United Arab Emirates, is consistently above 80%. In addition to sex parameters, SLE is also an age-related disease. Based on the study by Aguirre et al. [18], the average age of patients with SLE in this study was younger than the average age of the Asian race. The mean age in this research also differs from the neighboring country, Malaysia. According to Shaharir et al. [19], the mean age of patients with SLE in Malaysia was 40.3 ± 14.4, nine years apart from the mean age in this study. This is also in line with Hamijoyo et al. [20], who discovered that the average age of lupus patients in Indonesia was 27.7 ± 9.4. As a result, Indonesia's lupus patients are relatively younger compared to other Asian countries.

The elevated SLE disease activity was indicated by an increase in the SLAM score, which plays a central role in assessing outcomes and differences between groups of patients with SLE [21, 22]. Based on their SLAM scores, all patients in this study had severe disease activity. We found that the serum complement level decreased as the SLAM score increased. These results align with several other studies that found low complement levels in severe SLE [23–25]. The activation of the complement pathway in patients with SLE causes the continuous use of complements and decreases the serum levels of C3 and C4. Nevertheless, the use of complement as a biomarker is still controversial. The univariate analysis of C3 and C4 levels shows a decrease during the renal flare, not two months before. On the other hand, the multivariate regression analysis shows that two months before the onset of the renal flare, the C4 levels dropped significantly (but not the C3 levels) along with other clinical factors such as younger age and increased ESR [26].

We also found significant complement differences between the groups with high and normal leukocyte counts, suggesting that the role of leukocytes in SLE activity may be related to complements. Supported by Birmingham and Hebert [27], infiltrating leukocytes are attached to the complement proteins via leukocyte complement receptors, resulting in the release of proinflammatory mediators and tissue-damaging factors. Anaphylatoxins are also released by activated complements and bind to leukocyte complement receptors, causing activation and chemotaxis to the inflammatory tissue sites and leading to the pathogenesis and tissue injury of SLE.

The reciprocal interaction happens between the complement system and proinflammatory cytokines [28]. Complement is an essential part of innate immunity that protects the host from infection by pathogens. Activated complement eliminates pathogens by opsonization through activated C3 and C4, direct lysis by a membrane attack complex, and stimulation and activation of innate immune cells by producing the anaphylatoxin [29, 30]. Several reports suggest that proinflammatory cytokines enhance the expression of anaphylatoxin receptors in inflammatory cells [31, 32]. Also, the effect of anaphylatoxin on cytokine expression appears to be strictly dependent on the pathophysiological status of the ongoing inflammatory response. On the other hand, given the potentially destructive role of Th17 cells in autoimmune diseases, complement interacts with toll-like receptors (TLRs) and promotes Th17 cells, especially in the context of pathogen infections, resulting in autoimmune and inflammatory tissue damage. But the mechanisms of many infection-related autoimmune diseases remain poorly defined. Crosstalk and amplification between complement and TLR in the context of microbial infection facilitate priming and proliferation of self-reactive Th17 via molecular mimicry [33].

The cytokine levels in the present study were increased in patients with higher SLE activity. Although not statistically significant, cytokine levels in patients with SLE in the present study tended to increase along with disease activity. Cytokine activation is essential in the pathogenesis of autoimmune diseases such as SLE and lupus nephritis. The inflammatory cytokine IL-6 levels have been reported to correlate with SLE disease activity, and urinary IL-6 levels were elevated in patients with proliferative lupus nephritis with high levels of anti-dsDNA antibody [23, 34]. The IL-6 also interacts with TGF-*β* and promotes T-helper 17 (Th17) cell differentiation, which drives further inflammation and exacerbates autoimmune disease [35]. Supporting our findings and the proposed mechanism, Ali et al. [36] discovered disturbances in serum IL-17 and TGF-*β* levels in patients with SLE compared to the healthy controls, but the IL-17/TGF-*β* ratio was not significantly associated with disease activity.

The TGF-*β* levels were significantly higher in patients with high creatinine levels than in patients with normal creatinine. Despite being insignificant, TGF-*β* levels were also higher in patients with higher BUN levels. Similar findings were found in Paradowska-Gorycka et al. [15] research, which discovered an increase in creatinine in patients with high TGF-*β* levels; the study also stated that TGF-*β* significantly impacts both susceptibility to SLE and the more active stage of the disease through the influence of HLA-DRB1*∗*52.1 expression.

The proportion of participants who develop end-organ disease in SLE may vary, but renal and hematological disorders are the predominant manifestations [18]. However, many factors influence organ damage, including age, disease duration, gender, ethnicity, disease activity, corticosteroid usage, poverty, hypertension, and abnormal disease behavior [22, 37]. Although all patients have a severe SLE, their renal damage tends to be low, as indicated by normal BUN and creatinine levels. However, moderate proteinuria was found in the majority of patients with SLE in this study which suggests that impaired renal filtration has occurred. Previous studies have shown proteinuria to be an important marker of kidney damage and lupus nephritis, even at low levels. Rosa et al. [38] suggested that patients with SLE may have significant renal pathology with low proteinuria. In addition, Chedid et al. [39] stated that low-grade proteinuria might have a significant association with lupus-associated or nonassociated kidney disease.

Hematological symptoms are also common in patients with SLE. Research by Eilertsen et al. [40] is concordant with our finding of a significant increase in IL-6 in patients with higher ESR. Although there is no clear explanation of the pathway or mechanism relating ESR and IL-6, the increase in ESR occurs due to changes in serum proteins, mainly fibrinogen, triggered by proinflammatory factors [41]. Carty et al. [42] found that inflammatory IL-6 is a primary regulator of fibrinogen synthesis through its interaction with fibrinogen genes (FGA, FGB, and FGG). IL-6 binds to its receptor and activates the STAT3 protein in the intracellular pathway, which triggers response elements in the promoter region of fibrinogen to provide transcription [43–45].

The limitation of the current study is the small number of patients and the single-center study. A larger number of participants, including those receiving a different treatment, should be examined to serve a broader perspective on the association of complement and cytokines with SLE disease activity. Furthermore, ethnic linkages in SLE make more comprehensive cytokine and immune cell profiles prominent and should be validated to fully understand the immune mechanisms underlying disease pathogenesis, particularly in the patients with SLE population in Indonesia. A cytokine profile assessment, supported by complements evaluation, may become the suitable marker for better diagnosing and treating patients with naïve SLE.

## 5. Conclusions

From the results of this study, based on the SLAM score, we found all patients with naïve SLE in Dr. Soetomo general hospital were severe. There is no significant association between SLAM and renal impairment or hematologic activity, but patients with higher SLAM had a significant decrease in complement; this complement increase was also significant in patients with higher leukocyte counts. Although not statistically significant, an increase in cytokines was also observed in patients with higher SLAM. Patients with high serum creatinine had a significant increase in TGF-*β*, whereas patients with a faster ESR also had a significant increase in IL-6. Larger-scale research is needed to better understand cytokine alteration and its role in the pathogenesis of SLE, especially in the Indonesian population. Although kidney damage and hematological activity are not necessarily the primary manifestation of patients with SLE in this research, but assessment of cytokine profiles, in conjunction with complements evaluation, may become a promising marker for reliable diagnosis and treatment of SLE in the future.

## Figures and Tables

**Table 1:** Patient's characteristics.

Characteristics (*n*/mean ± SD/median (95% CI))	
Sex (female : male)	30 : 0
Age	31.3 ± 10.5 years
SLAM score	29.30 ± 3.88
BUN	14 (11–19) mg/dL
Creatinine	0.725 (0.54–1.20) mg/dL
Proteinuria	
Unremarkable (0)	5
Moderate (+1 and +2)	19
Severe (+3 and +4)	6
Leukocyte	8056 ± 3442 cells/*μ*L
Platelet	190500 (52000–305000) cells/*μ*L
Hematocrit	21.96 ± 6.578%
ESR	61.83 ± 38.18 mm/hour
Hemoglobin	7.13 ± 2.32 g/dL
Lymphocyte	967.9 ± 427.8 cells/*μ*L
CRP	7.84 (3.20–26.21) mg/L
C3	28.88 ± 12.98 mg/dL
C4	15.25 (7.60–20.60) mg/dL
TGF-*β*	35 ± 7.02 ng/mL
IL-6	200.6 (127.6–300.5) pg/mL
Anti-dsDNA	155.32 ± 186.10 IU/mL
ANA test	86.61 (65.50–184.6)

SLAM, systemic lupus activity measurement; BUN, blood urea nitrogen; ESR, erythrocyte sedimentation rate; CRP, C-reactive protein; C3, complement 3; C4, complement 4; TGF-*β*, transforming growth factor-beta; IL, interleukin; dsDNA, double-stranded DNA; ANA, antinuclear antibody.

**Table 2:** Association of disease activity with renal damage and hematological activity.

Correlations	*r*	*p*
SLAM × BUN levels	0.035	0.856
SLAM × creatinine levels	0.082	0.666
SLAM × proteinuri	−0.017	0.929
SLAM × leukocyte	0.283	0.129
SLAM × platelet	0.348	0.060
SLAM × hematocrit	0.269	0.151
SLAM × ESR	−0.189	0.318

SLAM, systemic lupus activity measurement; BUN, blood urea nitrogen; ESR, erythrocyte sedimentation rate.

**Table 3:** Association of complement and cytokine levels with renal damage and hematological activity.

	*A*	*B*	Statistical significance
BUN level	**5–20 mg/dL (*n*** **=** **21)**	**>20 mg/dL (*n*** **=** **9)**	
C3	27.04 ± 11.19	33.18 ± 16.39	ns
C4	15.00 (7.60–20.60)	16.10 (6.00–36.00)	ns
TGF-*β*	34.44 ± 6.67	36.79 ± 7.96	ns
IL-6	194.5 (94.42–242.9)	358.5 (79.20–585.6)	ns

Creatinin level	**≤1.2 mg/dL (*n*** **=** **21)**	**>1.2 mg/dL (*n*** **=** **9)**	
C3	28.60 ± 12.41	29.56 ± 15.01	ns
C4	15.50 (7.6–20.9)	14.30 (6–25.6)	ns
TGF-*β*	33.48 ± 6.79	39.04 ± 6.241	*p*=0.0445
IL-6	206.8 (94.42–300.5)	162.4 (79.20–585.6)	ns

Proteinuria	**0 and +1 (*n*** **=** **17)**	**+2, +3, +4 (*n*** **=** **13)**	
C3	28.19 ± 12.45	29.78 ± 14.11	ns
C4	13.90 (7–20.6)	15.80 (6.6–25.6)	ns
TGF-*β*	34.77 ± 7.12	35.64 ± 7.15	ns
IL-6	194.5 (120.4–300.5)	229.2 (79.20–536.0)	ns

Leukocyte count	**≤11000 cells/ ** *μ * **L (*n*** **=** **24)**	**>11000 cells/ ** *μ * **L (*n*** **=** **6)**	
C3	31.91 ± 12.49	16.77 ± 6.287	*p*=0.0081
C4	16.45 (10–24.4)	6.00 (5–15.5)	*p*=0.0027
TGF-*β*	33.38 ± 6.326	42.22 ± 5.191	ns
IL-6	210 (94.42–358.5)	178 (16.19–860.5)	ns

Platelet count	**150–400 × 10 ** ^**9**^ ** /L (*n*** **=** **15)**	**150–400 × 10 ** ^**9**^ ** /L (*n*** **=** **15)**	
C3	29.81 ± 13.96	27.96 ± 12.35	ns
C4	14.30 (7.00–25.60)	15.80 (7.00–20.90)	ns
TGF-*β*	37.06 ± 5.531	33.24 ± 7.982	ns
IL-6	168.5 (79.20–406.6)	213.8 (120.4–433.5)	ns

Hematocrit	**35–25% (*n*** **=** **11)**	**<25% (*n*** **=** **19)**	
C3	28.73 ± 8.60	28.97 ± 15.18	ns
C4	16.10 (7.600–23.50)	14.30 (6.000–25.60)	ns
TGF-*β*	33.77 ± 6.608	35.94 ± 7.305	ns
IL-6	213.8 (94.42–406.6)	168.5 (75.03–517.7)	ns

ESR	**≤29 mm/hour (*n*** **=** **7)**	**>29 mm/hour (*n*** **=** **23)**	
C3	33.53 ± 11.29)	27.47 ± 13.21	ns
C4	24.40 (6.60–34.20)	14.30 (7.60–18.00)	ns
TGF-*β*	37.19 ± 5.88	34.52 ± 7.34	ns
IL-6	89.57 (18.73–300.5)	213.8 (161.5–433.5)	*p*=0.0327

C3, complement 3; C4, complement 4; TGF-*β*, transforming growth factor-beta; IL, interleukin; ESR, erythrocyte sedimentation rate; ns: no significance. Bold values indicated the grouping parameters of groups A and B.

## Data Availability

All data used to support the findings of this study are available from the corresponding author upon request.
